# Predictors of major adverse events and complications after ventricular septal defects surgical closure in children less than 10 kg

**DOI:** 10.1186/s13019-022-01985-6

**Published:** 2022-09-07

**Authors:** Ayman R. Abdelrehim, Mustafa Al-Muhaya, Alassal A. Alkodami, Luna S. Baangood, Mansour Al-Mutairi, Abdul Quadeer, Fath A. Alabsi, M. Alashwal, Mohamed Mofeed F. Morsy, Abdulhameed A. Alnajjar, Sherif S. Salem

**Affiliations:** 1 Pediatric Cardiac Services, Madinah Cardiac Center MCC, Madinah, Saudi Arabia; 2 Cardiothoracic Surgery Department, Faculty of Medicine, Menoufia University, Shebeen El-Kom, Menoufia Egypt; 3 Pediatric Department, Faculty of Medicine, Menoufia University, Shebeen El-Kom, Menoufia Egypt; 4 Pediatric Department, Faculty of Medicine, Sohag University, Sohag, Egypt

**Keywords:** Ventricular septal defect, Surgical closure, Major adverse events

## Abstract

**Background:**

Ventricular septal defect (VSD) is the most common congenital cardiac defect for which outcomes are not uniform. There is a lack of consensus on the risk factors for the unfavorable outcomes following surgical VSD closure.

**Aim:**

The aim of this study was to determine the risk factors and the predictors of major adverse events (MAEs) and complications following surgical closure of VSD in children weighing less than 10 kg.

**Methods:**

This retrospective cohort study included children less than 10 kg who underwent surgical closure of congenital VSD of any type with or without associated congenital heart diseases. Patients with associated major cardiac anomalies were excluded. Preoperative, operative and postoperative data were collected from medical records.

**Results:**

This study included 127 patients 52.8% were males, the median age was 8.0 months (IQR = 6.0–11.0 months), and their median weight was 5.7 kg (IQR = 4.8–7.0). Mortality was in one patient (0.8%) Multivariable logistic regression analysis revealed that male sex group (observational data), previous pulmonary artery banding (PAB), and significant intraoperative residual VSD were significant risk factors for the development of MAEs (odds ratios were 3.398, 14.282, and 8.634, respectively). Trisomy 21 syndrome (odds ratio: 5.678) contributed significantly to prolonged ventilation. Pulmonary artery banding (odds ratio: 14.415), significant intraoperative (3 mm) residual VSD (odds ratio: 11.262), and long cross-clamp time (odds ratio: 1.064) were significant predictors of prolonged ICU stay, whereas prolonged hospital stay was observed significantly in male sex group (odds ratio: 12.8281), PAB (odds ratio: 2.669), and significant intraoperative (3 mm) residual VSD (odds ratio: 19.551).

**Conclusions:**

Surgical VSD repair is considered a safe procedure with very low mortality. Trisomy 21 was a significant risk factor for prolonged ventilation. Further, PAB, significant intraoperative residual of 3 mm or more that required a second pulmonary bypass, and a greater cross-clamp time were significant predictors of MAE and associated complications with prolonged ICU and hospital stay.

## Introduction

Surgical repair of VSD is the most commonly performed pediatric cardiac procedure worldwide. However, the surgical outcomes are not uniform globally [26]. Surgical intervention is reserved for larger defect sizes, greater left ventricular volume overload, and pulmonary hypertension [5].

Surgical repair of VSD is usually very effective, and advances in surgical techniques and in postoperative patient care have led to a decrease in postoperative mortality and morbidity. Though, risks of a complicated course and death still exist [8, 15].

Worldwide, there is a deficiency in consensus regarding the risk factors for the complicated and unfavorable outcomes following surgical VSD closure [9]. Some studies linked the prognosis to the anatomic location and size of the defect [12], age at operation [19], the volume of the shunt and pulmonary artery pressure, accompanying cardiac lesions, and previous surgical intervention [6]. Other studies found that low weight at the time of the operation and age less than 6 months is associated with postoperative complications [1].

Estimation of the prognosis in congenital heart diseases including VSD is of great value to patients, their families, and the physicians. Information about the prognostic predictors of outcomes helps to make realistic expectations, optimizes patients’ selection for surgery, and provides a standard for comparing the outcomes of different proposed therapies [7].

Therefore, The aim of to determine the risk factors and the predictors of major adverse events (MAEs) and complications following surgical closure of VSD in children weighing less than 10 kg. (MAEs) defined as significant residual VSD greater than 3 mm post-operative, unplanned second operations, complete heart block requiring a permanent pacemaker, pulmonary hypertension, deep sternal wound infection, renal impairment requiring dialysis, and/or death, and for the predictors of prolonged ventilation, intensive care unit (ICU), and hospital stay following surgical closure of VSD in children less than 10 kg.

## Methods

### Study design, settings, and ethical considerations

The study was approved by the Ethics Committee of Madinah Cardiac Center (Approval number 2021R23). Informed written consents were obtained from the patients ‘parents, or guardians. This retrospective cohort study was conducted at the Department of Pediatric Cardiac Surgery and Cardiology, Madinah Cardiac Center MCC Hospital from June 2016 through March 2022. Confidentiality of patients' data was maintained via giving a code number for every patient.

### Inclusion criteria

All children weight less than 10 kg who were scheduled for surgical closure of congenital VSD of any type were included. Patients who had associated congenital heart diseases including atrial septal defect, coarctation of the aorta, patent ductus arteriosus (PDA), pulmonary artery hypertension, and/ or pulmonary artery stenosis were also included.

### Exclusion criteria

We excluded patients who had major cardiovascular anomalies such as discordant atrioventricular, ventriculo-arterial connection, tetralogy of Fallot, double outlet right ventricle, and/ or pulmonary atresia.

### Data collection

The hospital records were reviewed. Preoperative data including sex, age and weight at the operation time, history of Trisomy 21 syndrome, previous pulmonary artery banding (PAB), and the pressure gradient around the band as well as the time between the banding procedure and the surgical interference for VSD, right ventricular hypertrophy, and the presence of second congenital heart defects were collected. The information concerning the surgical operation including the technique of surgical VSD closure whether interrupted or continuous, the type of used patch, the times of cross-clamp and cardiopulmonary bypass (CPB), and the existence of intraoperative 3 mm significant residual (that needed second bypass run) were also noted. The duration of mechanical ventilation and ICU stay, duration of inotropes used, the total in-hospital stay and the presence of short-term or long-term postoperative complications were recorded.

### Outcomes

1. Major adverse events (MAEs) including significant postoperative VSD residual larger than 3 ml, complete heart block that needed a permanent pacemaker insertion, the need for unplanned second operations, postoperative pulmonary hypertension, deep sternal wound infection, renal dysfunction that required temporary renal peritoneal dialysis, and/or death.
2. Associated complications (as our center protocol)
  Prolonged ventilation (more than 24 h).
  Prolonged ICU stay (more than 3 days).
  Prolonged hospital stays (more than 7 days).

### Surgical procedures

In all patients, surgical procedures were accomplished through median sternotomy under CPB with cannulation of both the superior-inferior vena cava and mild-to-moderate systemic hypothermia. Intermittent cold blood cardioplegia was used for myocardial preservation. None of the cases required total circulatory arrest. The VSD was accessed through right atriotomy, then the location and borders of VSD were examined to decide whether to perform patch closure of VSD in interrupted or continuous fashion according to the surgeon preference. In the interrupted technique, the VSDs have closed with interrupted sutures all around. In continuous suturing the patch closure of VSD was accomplished through continuous suturing all around the VSD, with shallow suturing at the postero-inferior rim of the VSD. In patients with pulmonary artery stenosis or previous PAB, debanding of the pulmonary artery with pulmonary artery reconstructions was performed while the aortic cross-clamp was in situ. The tricuspid valve regurgitation was tested by filling the right ventricle with saline before the closure of the right atriotomy, and no patients required any intervention to the tricuspid valve. For detection of residual VSD, transesophagial echocardiography was done for patients weighing more than 5 kg. Instead, transepicardial echocardiography was done in patients weighing less than 5 kg to avoid esophageal damage. In our protocol, if there is a residual VSD of more than 3 mm will go for a second run bypass to revise and to repair the residual. In patients with concomitant PDA, it was ligated after starting CPB and before clamping the aorta.

### Statistical analysis

Statistical analysis and presentation of data were conducted by SPSS (Statistical Package for the Social Sciences) software computer program, version 22. We presented categorical data as numbers and percentages, whereas continuous data were tested for normality by the Shapiro–Wilk test, and they were presented as the median and interquartile range (25th–75th percentiles) because of non-normal distribution. Chi-Square, Fishers’ Exact, and the Mann–Whitney U tests were applied for the Univariate analysis as appropriate. For each outcome, significant variables, with a *p* value less than 0.05, in the univariate analysis were entered into a multivariable logistic regression analysis to determine the significant predictors of the MAEs, the prolonged ventilation, and the prolonged ICU and hospital stay. *p* values less than 0.05 were considered statistically significant.

## Results

**Fig. 1:**
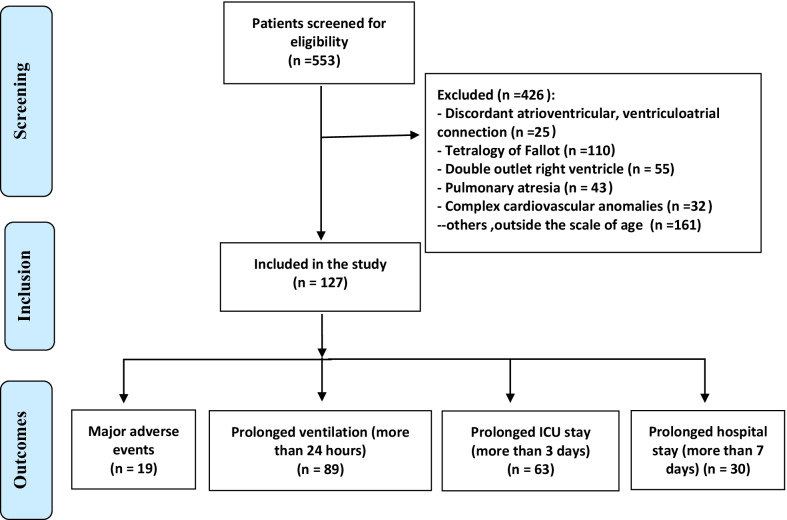
Patients selection flow chart

This study included 127 eligible patients who underwent surgical VSD closure as illustrated in the patient selection flow chart (Fig. 1). More than half (52.8%) were males, their median age was 8.0 (IQR = 6.0–11.0) months, and their median weight was 5.7 (IQR = 4.8–7.0) Kg. The most common type of VSD was inlet (34.6%), followed by an outlet (23%),Perimembranous types (23.6%) and the multiple VSD (5.5%). Thirty-nine (30.7%) patients were Trisomy 21 syndrome, and 59.1% had other associated congenital heart diseases. Right ventricular hypertrophy was recorded in 26 patients and PAB was done in 26 patients as well. The VSD repair was done by interrupted suture technique in (55.9%) and Gortex patch was used in (91.3%) of the patients. The presence of intraoperative (significant) residual VSD was found in 14 patients (11.0%). The median cross-clamp time was 60.0 (49.0–75.0) minutes, and the median total CPB time was 84.0 (71.0–102.0) minutes (Table 1).

**Table 1:** Baseline demographic and operative characteristics of the studied patients (n = 127)

Sex	Female	60 (47.2%)
	Male	67 (52.8%)
Age at operation (Months)	Minimum–Maximum	2.0–24.0
	Median (IQR)	8.0 (6.0–11.0)
Weight at operation (Kg)	Minimum–Maximum	2.9–10.0
	Median (IQR)	5.7 (4.8–7.0)
Type of VSD	Inlet	44 (34.6%)
	Outlet	30 (23.6%)
	Perimembranous	30 (23.6%)
	Multiple VSD	7 (5.5%)
	Others	16 (12.6%)
Trisomy 21 syndrome	Yes	39 (30.7%)
Associated CHD	Yes	75 (59.1%)
Right ventricular hypertrophy	Yes	26 (20.5%)
Pulmonary artery banding	Yes	26 (20.5%)
Type of VSD repair	Interrupted	71 (55.9%)
	Continuous	56 (44.1%)
Type of the used Patch	Gortex	116 (91.3%)
	Bovine	11 (8.7%)
The presence of intraoperative (Significant) residual VSD	Yes	14 (11.0%)
Cross clamp time (Minutes)	Minimum–Maximum	20.0–187.0
	Median (IQR)	60.0 (49.0–75.0)
Total CPB time (Minutes)	Minimum–Maximum	40.0–226.0
	Median (IQR)	84.0 (71.0–102.0)

*IQR* interquartile range, *VSD* ventricular septal defect, *CHD* congenital heart disease

Table 2 shows the postoperative outcomes of the studied patients. Patients who developed MAE accounted for 19/127 (14.3%). Twelve (9.4%) developed postoperative pulmonary hypertension, and 11(8.7%) developed renal dysfunction that required temporary renal peritoneal dialysis. Six (4.7%) had a significant post-operative VSD residual larger than 3 ml, and 7 (5.5%) patients required an unplanned second operation. Other observed MAEs included complete heart block that required permanent pacemaker implantation (3.9%), deep sternal wound infection (3.1%), and death (0.8%). Eighty-nine (70.1%) patients needed prolonged ventilation for more than 24 h, and about half (49.6%) stayed in ICU for more than 3 days. Prolonged hospital stay for more than 7 days was recorded in 30 (23.6%) cases.

**Table 2:** Incidence of major adverse events, prolonged ventilation, ICU, and hospital stays among the studied patients (n = 127)

	n = 127	%
Significant VSD residual larger than 3 ml (post-operative)	6	4.7
Unplanned second operation	7	5.5
Deep sternal wound infection	4	3.1
Renal dysfunction that required dialysis	11	8.7
Postoperative pulmonary hypertension	12	9.4
Complete heart block requiring a PPM implantation	5	3.9
Death	1	0.8
Major Adverse Events (All)	19	14.3
Prolonged ventilation (more than 24 h)	89	70.1
Prolonged ICU stay (more than 3 days)	63	49.6
Prolonged hospital stay (more than 7 days)	30	23.6

*VSD* ventricular septal defect, *ICU* intensive care unit, *PPM* permanent pacemaker

Table 3 demonstrates a univariate and multivariable logistic regression analysis for the MAEs. Male sex, greater age, and weight at operation time, the presence of right ventricular hypertrophy, tight (more than 60 pressure gradient) PAB, significant intraoperative residual, and higher cross-clamp time were significantly associated with the development of MAEs (*p* < 0.05). Multivariable logistic regression analysis revealed that PAB and significant intraoperative residual were significant risk factors for the development of MAEs (odds ratios were 3.398, 14.282, and 8.634, respectively).

**Table 3:** Univariate analysis and multivariable regression analysis for the factors associated with the development of major adverse events

Major adverse events						
Univariate analysis						*p* value
Sex	Female	51	52.0	9	31.0	0.047*
	Male	47	48.0	20	69.0	
Age at operation	Median (IQR)	8.0 (6.0–10.0)		9.0 (7.0–14.0)		0.020*
Weight at operation	Median (IQR)	5.5 (4.7–6.8)		6.0 (5.3–8.0)		0.045*
Down Syndrome	No	70	71.4	18	62.1	0.337
	Yes	28	28.6	11	37.9	
Type of VSD	Others	12	12.2	4	13.8	0.682
	Inlet	36	36.7	8	27.6	
	Outlet	23	23.5	7	24.1	
	PM	23	23.5	7	24.1	
	Multiple	4	4.1	3	10.3	
Associated CHD	No	44	44.9	8	27.6	0.132
	Yes	54	55.1	21	72.4	
Right ventricular hypertrophy	No	88	89.8	13	44.8	< 0.001*
	Yes	10	10.2	16	55.2	
Pulmonary artery banding	No	89	90.8	12	41.4	< 0.001*
	Yes	9	9.2	17	58.6	
VSD Repair	Interrupted	51	52.0	20	69.0	0.107
	Continuous	47	48.0	9	31.0	
Significant intraoperative Residual VSD	No	93	94.9	20	69.0	< 0.001*
	Yes	5	5.1	9	31.0	
Cross clamp time (Minutes)	Median (IQR)	59.5 (49.0–70.0)		69.0 (49.0–85.0)		0.086
Total CPB time (Minutes)	Median (IQR)	83.0 (70.0–93.0)		102.0 (77.0–140.0)		0.006*

Multivariable logistic regression analysis				
Risk factors	Beta coefficient	*p* value	Odds ratio	95% CI Odds ratio
Sex (male)	1.223	0.034*	3.398	1.098–10.516
Pulmonary artery banding	2.659	< 0.001*	14.282	4.722–43.195
Significant intraoperative (3 mm) residual VSD	2.156	0.003*	8.634	2.130–35.001

*IQR* interquartile range, *VSD* ventricular septal defect, *CHD* congenital heart disease, *CPB* cardiopulmonary bypass;

^*^significant at *p* < 0.05

Table 4 shows that prolonged ventilation was significantly associated with male sex, old age, Trisomy 21 syndrome, the presence of right ventricular hypertrophy, PAB, significant intraoperative residual, and higher CPB and cross-clamp times (*p* < 0.05). The prognostic risk factors for prolonged ventilation were Trisomy 21 syndrome (odds ratio: 5.678), and long Cross clamp time (odds ratio: 1.056).

**Table 4:** Univariate analysis and multivariable regression analysis for the factors associated with prolonged ventilation

Prolonged ventilation						
Univariate analysis						*p* value
Sex	Female	24	63.2	36	40.4	0.019*
	Male	14	36.8	53	59.6	
Age at operation	Median (IQR)	7.0 (5.5–8.0)		9.0 (7.0–11.0)		0.004*
Weight at operation	Median (IQR)	5.3 (4.7–6.3)		6.0 (5.0–7.0)		0.071
Down Syndrome	No	34	89.5	54	60.7	< 0.001*
	Yes	4	10.5	35	39.3	
Type of VSD	Others	4	10.5	12	13.5	0.369
	Inlet	9	23.7	35	39.3	
	Outlet	12	31.6	18	20.2	
	PM	11	28.9	19	21.3	
	Multiple	2	5.3	5	5.6	
Associated CHD	No	15	39.5	37	41.6	0.826
	Yes	23	60.5	52	58.4	
Right ventricular hypertrophy	No	35	92.1	66	74.2	< 0.001*
	Yes	3	7.9	23	25.8	
Pulmonary artery banding	No	36	94.7	65	73.0	0.006*
	Yes	2	5.3	24	27.0	
VSD Repair	Interrupted	20	52.6	51	57.3	0.627
	Continuous	18	47.4	38	42.7	
Significant intraoperative Residual VSD	No	37	97.4	76	85.4	0.039*
	Yes	1	2.6	13	14.6	
Cross clamp time (Minutes)	Median (IQR)	52.5 (41.0–60.0)		64.0 (53.0–79.0)		< 0.001*
Total CPB time (Minutes)	Median (IQR)	72.5 (63.0–83.0)		90.0 (78.0–115.0)		< 0.001*

Multivariable logistic regression analysis				
Risk factors	Beta coefficient	*p* value	Odds ratio	95% CI Odds ratio
Age at operation time	0.130	0.075	1.139	0.987–1.314
Trisomy 21 syndrome	1.737	0.005*	5.678	1.709–18.859
Cross clamp time	0.055	< 0.001*	1.056	1.027–1.087

*IQR* interquartile range, *VSD* ventricular septal defect, *CHD* congenital heart disease, *CPB* cardiopulmonary bypass;

^*^significant at *p* < 0.05

The prolonged ICU stay was significantly associated with the presence of right ventricular hypertrophy, PAB, significant intraoperative residual, and higher CPB and cross-clamp times (*p* < 0.05). Further, pulmonary artery banding (odds ratio: 14.415), significant intraoperative (3 mm or more) residual (odds ratio: 11.262), and cross-clamp time (odds ratio: 1.064) were significant predictors of prolonged ICU stay (Table 5).

**Table 5:** Univariate analysis and multivariable regression analysis for the factors associated with a prolonged ICU stay

Prolonged ICU stay						
Univariate analysis						*p* value
Sex	Female	34	53.1	26	41.3	0.181
	Male	30	46.9	37	58.7	
Age at operation	Median (IQR)	8.0 (6.0–10.0)		9.0 (6.0–12.0)		0.244
Weight at operation	Median (IQR)	5.5 (4.8–7.0)		5.7 (4.8–7.0)		0.739
Down Syndrome	No	49	76.6	39	61.9	0.073
	Yes	15	23.4	24	38.1	
Type of VSD	Others	8	12.5	8	12.7	0.889
	Inlet	20	31.3	24	38.1	
	Outlet	16	25.0	14	22.2	
	PM	17	26.6	13	20.6	
	Multiple	3	4.7	4	6.3	
Associated CHD	No	29	45.3	23	36.5	0.313
	Yes	35	54.7	40	63.5	
Right ventricular hypertrophy	No	60	93.8	41	65.1	0.022*
	Yes	4	6.3	22	34.9	
Pulmonary artery banding	No	62	96.9	39	61.9	< 0.001*
	Yes	2	3.1	24	38.1	
VSD Repair	Interrupted	32	50.0	39	61.9	0.177
	Continuous	32	50.0	24	38.1	
Significant intraoperative Residual VSD	No	63	98.4	50	79.4	0.001*
	Yes	1	1.6	13	20.6	
Cross clamp time (Minutes)	Median (IQR)	55.0 (45.0–63.0)		65.0 (52.0–84.0)		< 0.001*
Total CPB time (Minutes)	Median (IQR)	80.0 (68.0–87.0)		93.0 (80.0–124.0)		< 0.001*

Multivariable logistic regression analysis				
Risk factors	Beta coefficient	*p *value	Odds ratio	95% CI Odds ratio
Pulmonary artery banding	2.668	0.001*	14.415	3.031–68.547
Significant intraoperative (3 mm) residual VSD	2.421	0.034*	11.262	1.203–105.38
CPB time	-0.050	0.064	0.915	0.905–1.003
Cross clamp time	0.062	0.0091*	1.064	1.016–1.114

*ICU* intensive care unit, *IQR* interquartile range, *VSD* ventricular septal defect, *CHD* congenital heart disease, *CPB* cardiopulmonary bypass;

^*^significant at *p* < 0.05

The factors associated with prolonged hospital stay were the presence of right ventricular hypertrophy, PAB, significant intraoperative residual, and higher CPB and cross-clamp times (*p* < 0.05), whereas the significant predictors of prolonged hospital stay were male sex (odds ratio: 12.8281), PAB (odds ratio: 2.669), and significant intraoperative (3 mm or more) residual (odds ratio: 19.551) as shown in Table 6.

**Table 6:** Univariate analysis and multivariable regression analysis for the factors associated with a prolonged hospital stay

Prolonged hospital stay						
Univariate analysis						*p* value
Sex	Female	54	55.7	6	20.0	0.001*
	Male	43	44.3	24	80.0	
Age at operation	Median (IQR)	8.0 (6.0–10.0)		9.0 (7.013.0)		0.086
Weight at operation	Median (IQR)	5.5 (4.7–6.8)		6.0 (5.0–8.0)		0.297
Down Syndrome	No	70	72.2	18	60.0	0.207
	Yes	27	27.8	12	40.0	
Type of VSD	Others	11	11.3	5	16.7	0.806
	Inlet	34	35.1	10	33.3	
	Outlet	22	22.7	8	26.7	
	PM	25	25.8	5	16.7	
	Multiple	5	5.2	2	6.7	
Associated CHD	No	44	45.4	8	26.7	0.069
	Yes	53	54.6	22	73.3	
Right ventricular hypertrophy	No	86	88.7	15	50.0	< 0.001*
	Yes	11	11.3	15	50.0	
Pulmonary artery banding	No	87	89.7	14	46.7	< 0.001*
	Yes	10	10.3	16	53.3	
VSD Repair	Interrupted	50	51.5	21	70.0	0.075
	Continuous	47	48.5	9	30.0	
Significant intraoperative Residual VSD	No	93	95.9	20	66.7	< 0.001*
	Yes	4	4.1	10	33.3	
Cross clamp time (Minutes)	Median (IQR)	58.0 (49.0–67.0)		73.5 (54.0–90.0)		0.004*
Total CPB time (Minutes)	Median (IQR)	83.0 (70.0–92.0)		110.0 (83–143)		< 0.001*

Multivariable logistic regression analysis				
Risk factors	Beta coefficient	*p* value	Odds ratio	95% CI Odds ratio
Sex (male)	2.552	0.001*	12.8281	2.990–55.030
Pulmonary artery banding	2.542	< 0.001*	2.669	3.790–42.544
Significant intraoperative (3 mm) residual VSD	2.973	< 0.001*	19.551	3.809–100.358

*IQR* interquartile range, *VSD* ventricular septal defect, *CHD* congenital heart disease, *CPB* cardiopulmonary bypass;

^*^significant at *p* < 0.05

## Discussion

Cardiac surgeons strive to remain challenged to decide the best patient parameters that minimize the risk of complications improve outcomes in their practice. Mandate a greater probability of successful outcomes [13]. Therefore, this study investigated children less than 10 kg who was operated on for patch VSD closure.

In this study, the incidence of MAEs alone was recorded in 19/127 (14.3%) patients. Previous studies with a comparable cohort reported various incidence rates. Schipper et al. [22] described a total complications rate of 15.6% and an MAE rate of 2.9%, while Ergün et al. [9] showed a total complications rate of 33.5% and an MAE rate of 5.9%.On the other hand, MAEs accounted for 5.3% of all patients investigated by Anderson et al. [1]. These studies defined a composite of unintended unplanned reoperation, heart block requiring a permanent pacemaker, or circulatory arrest, and death as the MAEs.

The current work revealed a death rate of 0.8%. This coincides with Schipper et al. [22] who showed a 30-day and in-hospital mortality of 0% and a late death rate during follow-up of 0.8%. Earlier work that evaluated the outcomes of patients who underwent surgical repair of isolated VSD reported early and late postoperative deaths of 1.4%. The researchers emphasized that all deaths occurred in infants less than 3 months [2].

In the current study, six (4.7%) patients had significant VSD residual larger than 3 ml, most of them was in the multiple VSD group. Previous studies showed various incidence rates of residual VSD ranging from 16 to 51%, and a rate of spontaneous closure of these residual defects during a three-year follow-up in 71% [22, 23]. The observed variation in the incidence of residual VSD is related to different times of follow-up as well as different echocardiographic modalities.

The incidence of complete heart block that required permanent pacemaker implantation was 3.9%. A study including children less than 18 years who were operated on for VSD reported a total incidence of an atrioventricular block of 7.7%, among these patients 1.9% required a pacemaker. They conveyed that low body weight less than 4 kg was a risk factor for complete heart block [24].

In the studied cohort, seven (5.5%) patients required an unplanned second operation. These unplanned readmissions may have a significant influence on the emotional and financial aspects of the families [18]. The reported figure in our study is in line with Azhar [3] who detected a 10.5% readmission rate within 30 days and 15.9% at one year following congenital heart surgeries at King Abdulaziz University Hospital, Jeddah, Saudi Arabia.

Bases on multivariable analysis PAB and intraoperative residual of 3 mm or more were significant risk factors for the development of MAEs and associated complications (odds ratios were 3.398, 14.282, and 8.634, respectively). Our finding suggests that low birth weight infants could be safely staged by pulmonary artery banding to avoid complications. There are conflicting results regarding the prognostic role of the weight and age of the patient. Anderson et al. [1] reported that in infants less than 6 months, low body weight was a significant risk factor for the development of postoperative complications, while infants older than 6 months did not show this significant relationship. Further, Ergün et al. [9] recently emphasized that the increased body weight has a significant effect in reducing the risk of MAEs. Alternatively, an earlier study by Kogon et al. [16] reported that the body weight did not affect the development of complications or the length of ICU or hospital stay. Similar results have been reported by Schipper et al. [22].

Regression analysis in this study also revealed that Trisomy 21 patients have an increased likelihood (odds ratio: 5.678) for prolonged mechanical ventilation. This might be attributed to respiratory and feeding difficulties, the need for re-intubation, and the increased risk of infection [11]. About one-fourth of Trisomy 21 infants have VSDs [25]. The effects of Trisomy 21 on the postoperative outcomes and complications following surgical VSD closure are inconsistent [21].

In Saudi Arabia we don’t do prenatal screening or abortion (recently started), with high proportion of Down patients in our population (more than 1.9 /1000 incidence rate) and we are receiving high percent from this patients, The Society of Thoracic Surgeons mortality risk model considers any chromosomal abnormality or syndrome a potential factor that increases the risk of operative mortality [14]. However, other studies have specified that Trisomy 21 is not a significant risk factor for mortality [10, 17]. Recently, Purifoy et al. [21] displayed that Trisomy 21 was significantly associated with prolonged hospital stay after repair of Tetralogy of Fallot.

Pulmonary artery banding was a significant risk factor for the development of MAEs and complications, prolonged ICU, and hospital stay. Pulmonary banding is an interim palliative procedure, which is considered for multiple VSDs or apical malformations to reduce pulmonary blood flow. Further, it has been shown that PAB might affect the outcomes [20]. Inohara et al. [13] stated that PAB should be considered cautiously, particularly with the recent acceptable results after one-stage operations at earlier ages and smaller body weights.

Furthermore, the presence of a significant intraoperative residual of 3 mm or more and a greater cross-clamp time was a significant predictor of MAEs and associated complications, prolonged ICU, and hospital stays. A comparable study reported the occurrence of intraoperative complications, and prolonged ventilation duration among the significant independent predictors of prolonged postoperative length of stay following surgical correction of congenital heart diseases [4]. Other studies linked prolonged ICU and hospital stay following surgical VSD closure to the low body weight at operation time [2, 22].

## Limitations

This study is limited by its retrospective and observational nature. Additionally, the small number of patients available for study restricted the power of our analysis.

## Conclusions

Our findings indicate that surgical VSD repair is a safe procedure with a very low mortality rate. The incidence of MAEs 14.3%. The age and weight at the time of the operation do not contribute significantly to MAEs or postoperative complications. Trisomy 21 was a significant risk factor for prolonged ventilation. Further, the presence of PAB, significant intraoperative residual of 3 mm or more, and a greater cross-clamp time were significant predictors of MAEs and complications, prolonged ICU, and long hospital stay.

## Data Availability

The datasets used and/or analyzed during the current study are available from the corresponding author on reasonable request.
